# Development of Electronic Nose as a Complementary Screening Tool for Breath Testing in Colorectal Cancer

**DOI:** 10.3390/bios15020082

**Published:** 2025-02-01

**Authors:** Chih-Dao Chen, Yong-Xiang Zheng, Heng-Fu Lin, Hsiao-Yu Yang

**Affiliations:** 1 Department of Family Medicine, Far Eastern Memorial Hospital, New Taipei City 220, Taiwan; cdchen0815@gmail.com; 2 Department of Public Health, National Taiwan University College of Public Health, Taipei 100, Taiwan; b10801012@ntu.edu.tw; 3 Division of Trauma, Department of Surgery, Far Eastern Memorial Hospital, New Taipei City 220, Taiwan; hengfu57@gmail.com; 4 Graduate Institute of Medicine, Yuan Ze University, Taoyuan City 320, Taiwan; 5 Institute of Environmental and Occupational Health Sciences, National Taiwan University College of Public Health, Taipei 100, Taiwan; 6 Innovation and Policy Center for Population Health and Sustainable Environment (Population Health Research Center, PHRC), National Taiwan University, Taipei 100, Taiwan; 7 Department of Environmental and Occupational Medicine, National Taiwan University Hospital, Taipei 100, Taiwan; 8 Department of Family Medicine, National Taiwan University Hospital Yunlin Branch, Yunlin 640, Taiwan

**Keywords:** colorectal cancer, volatile organic compounds, electronic nose, machine learning, breath testing, breathomics

## Abstract

(1) Background: Colorectal cancer is one of the leading causes of cancer-related death, while early detection decreases incidence and mortality. Current screening programs involving fecal immunological testing and colonoscopy commonly bring about unnecessary colonoscopies, which adds burden to healthcare systems. The objective of this study was to provide an assessment of the diagnostic performance of an electronic nose serving as a complementary screening tool to improve current screening programs in clinical settings. (2) Methods: We conducted a case–control study that included patients from a medical center with colorectal cancer and non-colorectal cancer controls. We analyzed the composition of volatile organic compounds in their exhaled breath using the electronic nose. We then used machine learning algorithms to develop predictive models and provided the estimated accuracy and reliability of the breath testing. (3) Results: We enrolled 77 patients, with 40 cases and 37 controls. The area under the curve, Kappa coefficient, sensitivity, and specificity of the selected model were 0.87 (95% CI 0.76–0.95), 0.66 (95% CI 0.49–0.83), 0.81, and 0.85. For subjects at an early stage of disease, the sensitivity and specificity were 0.90 and 0.85. Excluding smokers, the sensitivity and specificity were 0.88 and 0.92. (4) Conclusions: This study highlights the promising potential of breath testing using an electronic nose for enabling early detection and reducing unnecessary treatments. However, more independent data for external validation are required to ensure applicability and generalizability.

## 1. Introduction

Globally, colorectal cancer (CRC) is the third most common cancer and the second leading cause of cancer-related deaths worldwide [1], remaining a significant public health issue. In Taiwan, the number of CRC cases has consistently ranked first, and the age-adjusted CRC incidence and mortality are 46.77 and 14.46, exceeding those reported in other Asian countries [2,3]. The early detection of CRC through testing methods with appropriate subsequent treatments can reduce mortality rates [4], making the development of accurate, reliable, and effective screening tools a key priority to promote public health in CRC management.

Since 2004, the Ministry of Health and Welfare in Taiwan has promoted a screening program using fecal immunochemical testing (FIT). This program adopts a two-step approach, where individuals with positive FIT results are referred for confirmatory colonoscopy, a practice aligned with screening strategies in many other countries [3]. However, FIT has limitations, particularly in detecting early-stage CRC, as it misses approximately one-third of stage I cases. FIT demonstrated a sensitivity of 68% (95% CI: 57–78%) for stage I cancers, 92% (95% CI: 87–96%) for stage II cancers, 82% (95% CI: 73–89%) for stage III cancers, and 89% (95% CI: 80–95%) for stage IV cancers (*p* for trend = 0.01) [5]. Complementary tools are needed to improve the non-invasive detection of early-stage CRC.

The progression of disease changes the volatile organic compounds (VOCs) in human bodies [6,7]. Testing methods for screening CRC by analyzing VOCs have been proposed, including the use of VOCs in blood [8], urine [9,10,11], and feces [12,13,14] as biomarkers. Breathomics techniques, using gas chromatography-mass spectrometry (GC-MS) and gas sensor arrays, can identify the composition of volatile metabolites in exhaled breath, which may be used in screening for various diseases. Techniques for analyzing breath volatile biomarkers have been used to distinguish healthy individuals from those who with specific diseases [15,16,17], including CRC [18,19,20,21,22,23,24]. Compared to other biomarkers, breath testing is a less invasive, less costly, more comfortable, and faster method without limited samples and frequency in screening CRC [16,17,22,24]. These properties have made breath testing a promising testing method and may be suitable as a more acceptable additional test in clinical practice [18]. However, despite its potential, breath testing in CRC remains underdeveloped in terms of clinical validation. It is unclear how external factors such as diet, smoking, and other co-existing conditions may influence these profiles [22,25]. Therefore, further research is necessary to address these gaps. Developing standardized methodologies and validating the diagnostic accuracy and reliability of breath testing can facilitate its integration into current screening programs for CRC.

Electronic noses consist of gas sensor arrays that discern a specific VOC composition in combination with machine learning analysis, with each sensor array containing several individual sensors blended with a carbon black composite. As VOCs enter the electronic nose and attach to each sensor, the polymer film on the surface may start swelling and increase the electronic resistance of the sensor [26,27]. The electronic signals responding to the change in resistance can serve as features of specific VOC patterns, so-called “fingerprints” for different VOC compositions, and are able to build a predictive model through machine learning algorithms to detect diseases [26,28]. Due to its low cost, accessibility, ease of operation, and short analysis time [25,29], the electronic nose has been utilized for assessing food quality or potential hazardous gases in the environment [30]. In recent years, it has been validated for screening diseases such as pneumoconiosis [31], nosocomial infectious pneumonia [32], breast cancer [33], and for rapid COVID-19 detection [34]. Although previous studies have shown the potential of electronic noses for detecting CRC [21,22,24,25], limited research suggests whether breath testing established through electronic nose techniques and machine learning analyses can be supportively integrated into CRC screening programs.

The main objectives of this study are to evaluate the accuracy of the electronic nose in CRC screening at different stages and to assess its potential as a complementary tool to improve current screening programs in clinical settings.

## 2. Materials and Methods

The research protocol was approved by the Ethics Committee of the Far Eastern Memorial Hospital (Approval No. 110021-E). We obtained written informed consent from all study participants before the study. All methods in this study were conducted in accordance with relevant guidelines and regulations.

### 2.1. Study Design

**Figure 1:**
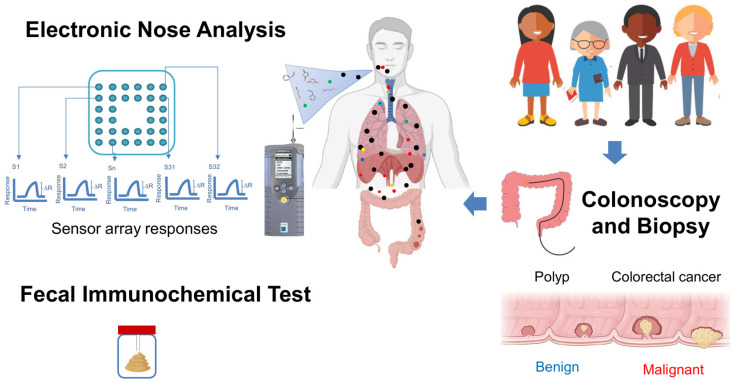
Overview of the study process and key technical details of electronic nose-based CRC screening.

We conducted a case-control study as shown in Figure 1. We collected human samples of exhaled breath at a medical center in New Taipei City from March 2023 to August 2024, which were analyzed at the College of Public Health of National Taiwan University.

### 2.2. Study Subjects

We recruited study participants from the gastrointestinal (GI) surgery and family medicine departments at the hospital. Prior to enrollment and sampling, participants were informed about the study’s purpose and given detailed instructions regarding the procedures and requirements. Participants who consented to join the study underwent colonoscopy and a biopsy was performed if polyps were detected. The following exclusion criteria were applied: individuals who (1) did not provide consent, (2) were outside the age range of 20 to 74 years, (3) were unable to effectively communicate or perform the exhaled breath sampling, (4) had poor daily functioning, (5) had a life expectancy of less than six months, or (6) were in the long-term care facility.

### 2.3. Study Variables

#### 2.3.1. Reference Standard

The diagnosis of CRC was based on pathological reports and colonoscopy results [35], which were the reference test for determining CRC cases and non-CRC controls in this study. Subjects diagnosed with CRC were classified as cases, and the pathological stage of the primary tumor was determined based on the Cancer Staging Manual [36] during a joint conference comprising GI surgeons, pathologists, and oncologists at the hospital. Subjects confirmed to be free of CRC or found to have benign polyps through biopsy were classified as controls. For ambiguous diagnoses, clinical reports were reviewed to finalize classification, while those with unresolved diagnoses would be excluded from this study. If the pathological stage was undetermined, the subject was included but reviewed in sensitivity analysis.

#### 2.3.2. Exhaled Breath Sampling

**Figure 2:**
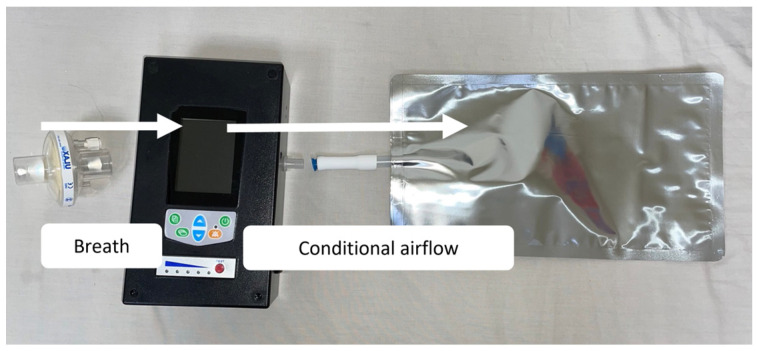
Exhaled breath sampling design. We used disposable single-use mouthpieces (bacterial/viral filter with HME, Ab ULAX, Motala, Sweden) and disposable single-use sampling bags (1000 mL aluminized gas collection bag, Hope Wang Enterprises, Taipei, Taiwan). In order to collect endogenous VOCs, we used a specific collector device that was developed by Professor Yao-Kun Li’s laboratory in the Department of Applied Chemistry at National Yang Ming Chiao Tung University. As the concentration of carbon dioxide reached the level that equates to alveoli air, the collector device began directing exhaled breath to flow into the air sampling bag connected to it at a fixed flow rate. The red flashing light on the device indicated that the directing process had started, allowing both the sampling personnel and the subject to confirm proper collection.

For ensuring physiological metabolic significance, we collected the VOCs from alveoli, preventing the potential influence of the subjects’ diet and the airflow of exhalation on the concentration of the VOCs [16,17]. Therefore, during sampling, we used a collector device that could instantly detect the concentration of carbon dioxide in the exhaled breath, ensuring that the sampled air would contain endogenous VOCs from the alveoli [16,17,37,38]. The sampling device was shown in Figure 2. The sampling process was conducted in the same office, free from other potential VOC sources and equipment that could drastically change the temperature or humidity in the room, ensuring that all subjects were sampled under identical environmental conditions. Subjects then held the mouthpiece with their mouths directly and started exhaling until the sampling bag was fully filled. The exhalation process took about 10 min, with the sampling personnel assisting beside. Subjects were able to take a short break and a deep breath before resuming exhalation if needed.

#### 2.3.3. Subjects’ Characteristics

We obtained the exposure variables of external factors relevant to this study, such as age; gender; and medical history of conditions including diabetes [39], chronic kidney disease [40], and chronic obstructive pulmonary disease (COPD) [41], which might influence the VOC composition of exhaled breath [22]. Additionally, we obtained information on cigarette smoking [42] and alcohol consumption [43] from the records provided by the hospital and they were included in the analysis. All variables were handled in compliance with privacy and ethical regulations, ensuring that the subjects’ personal information was protected with appropriate security and confidentiality.

### 2.4. Exhaled Breath Analysis

The sampled air was sent to the College of Public Health of National Taiwan University for analysis. Although studies have shown that VOCs in the bags used are stable at room temperature [37,39], to avoid any unforeseen factors, the samples were analyzed as soon as possible. We used an electronic nose, the Cyranose320 (Sensigent, Baldwin Park, CA, USA), which has 32 thin-film nanocomposite sensors, to analyze the exhaled breath. The electronic signals responding the change in the resistance after attaching to VOCs, including reaction time, recovery time, and voltage peak, were used to create fingerprints of the VOC composition in the exhaled breath through appropriate analytical methods, thereby discerning specific patterns of VOCs [29,44]. During measurement, a needle was inserted into the tube of the sampling bag and it was connected to the electronic nose with a PVC hose, fixed by a three-way valve. Additionally, the electronic nose was connected to a glass bottle containing silica gel desiccant to remove external moisture. The electronic nose was finally connected to the computer to record electronic signals. The electronic nose repeatedly measured each sample for 10 times, with air flow rate set at 120 mL/min. After each measurement, it underwent a 10 s baseline purge, a 40 s sample purge, and a 10 s wash-out. Since the electronic nose was sensitive to temperature and humidity, the measurement was conducted in a room where the environment temperature was controlled at an average of 22.6 °C (standard deviation 2.4 °C) and humidity at 55% (standard deviation 7%) [45].

### 2.5. Data Preprocessing

**Figure 3:**
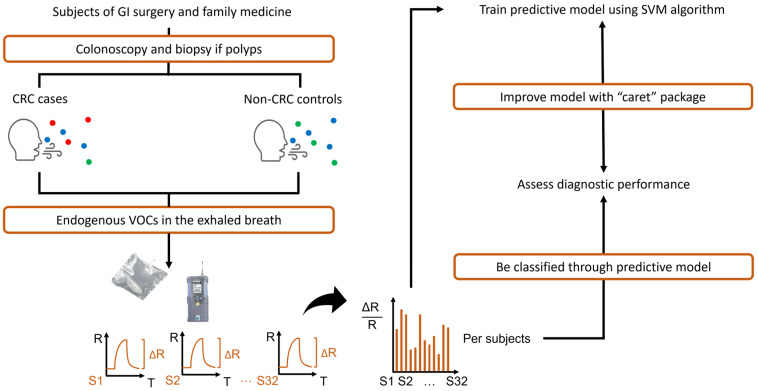
Study flow chart. This chart shows the design of this study, including the procedures from sampling to analyzing. CRC cases and non-CRC controls determined by pathological report and colonoscopy had different metabolic conditions and released different VOC compositions. After sampling of the exhaled breath, we analyzed them with an electronic nose and preprocessed the data. We used SVM algorithm to build predictive model and classified subjects. We finally assessed the diagnostic performance by estimating accuracy, Kappa coefficient, sensitivity, specificity, PPV, NPV, ROC curve, and AUC. CRC: colorectal cancer, FIT: fecal immunochemical test, VOCs: volatile organic compounds, R: resistance, ΔR: change in resistance, S: sensor, SVM: support vector machine.

The procedures used to establish the prediction model are shown in Figure 3. The signal values derived from the 32 sensors underwent the preprocessing involving normalization and standardization. The electronic nose first calculated the relative change as a response for each sensor, which divided the change in resistance by its original resistance. The normalized value represents the proportion of each sensor’s relative change within the overall change, which is a widely used method for values involving changes and suitable for processing the data derived from the sensor array of the electronic nose. We then subtracted the mean of 32 normalized values and divided it by the standard deviation to transform the values. This process of normalization and standardization adjusted the data so that the mean became 0 and the standard deviation became 1, with all the values ranging between 0 and 1, which effectively mitigated the influence of noise and outliers [46]. Missing data in signals from the electronic nose were implemented by the averaged value measured by the corresponding sensor across all subjects.

$$\mathrm{Sensor}\;\mathrm{response}\;(\mathrm{relative}\;\mathrm{change}):\;\Delta R/R_{0}$$

$$\mathrm{Normalization}:\;{\left(\Delta R/R_{0}\right)}_{i}\;/\;\sum |{\Delta R/R_{0}|}_{j}$$

$$\mathrm{Standardization}:\;\left(Normalized\;value-mean\right)/standard\;deviation$$

To obtain a representative value for each subject’s exhaled breath sample, each sensor in the electronic nose recorded 10 repeated measurements for each subject. The values from these repeated measurements were averaged into a single value for each sensor per subject. This averaged data for all 32 sensors were used in the subsequent statistical analyses, ensuring that the results reflect a more stable measurement for each subject. To further avoid the influence of data drift on modeling, we excluded sensor data S5 and S31 due to their production of exceptionally large values [27,45], using the remaining 30 preprocessed sensor data points as predictors in the statistical analyses.

### 2.6. Statistical Analysis

We used a supervised machine learning algorithm to build predictive models. Support vector machine (SVM) is an algorithm suitable for analyzing data derived from electronic noses [32,45,47]. SVMs create a boundary known as a hyperplane dividing the data space into relatively uniform sections on either side, and map data to a higher-dimensional space for better separability with the kernel function, represented as

$$K\left(\vec{x_{i}\;},\vec{x_{j}\;}\right)=\varphi (\vec{x_{i}\;})\times \varphi (\vec{x_{j}\;})$$

We used the “train” function in the “caret” package to perform the SVM algorithm including a linear kernel, a polynomial kernel, and a radial basis kernel for modeling.

$$\begin{array}{c} \mathrm{Linear}\;\mathrm{kernel}:\;K\left(\vec{x_{i}\;},\vec{x_{j}\;}\right)=\vec{x_{i}\;}\times \vec{x_{j}\;}\\ \mathrm{Polynomial}\;\mathrm{kernel}:\;K\left(\vec{x_{i}\;},\vec{x_{j}\;}\right)={\left(\vec{x_{i}\;}\times \vec{x_{j}\;}+1\right)}^{d}\\ \mathrm{Radial}\;\mathrm{basis}\;\mathrm{kernel}:\;K\left(\vec{x_{i}\;},\vec{x_{j}\;}\right)=e^{\frac{-{\left\|\vec{x_{i}\;}-\vec{x_{j}\;}\right\|}^{2}}{2\sigma^{2}}} \end{array}$$

We applied leave-one-out cross validation (LOOCV) for internal validation. In the resampling method of LOOCV, each data point was sequentially used as a validating set while the remaining data served as the training set, which was repeated for every single data point in the dataset, ensuring that the models were validated in each observation [48]. The “caret” package then helped select one model with the highest accuracy. After the predictive model was built, we used the same dataset as the testing set to classify the subjects as having CRC or not. With the reference standard of pathological reports and colonoscopy results, we estimated the overall performance indicators, including accuracy, sensitivity, specificity, positive predictive value (PPV), negative predictive value (NPV), receiver operating characteristic (ROC) curve, area under the ROC curve (AUC), and Kappa coefficient. Subjects with abnormal data derived from the electronic nose which resulted in indetermined classification were excluded.

An AUC value of 0.7–0.8, 0.8–0.9, or 0.9–1.0 indicates good, very good, or excellent diagnostic accuracy, respectively [33,49]. To account for the possibility of a correct prediction by chance, we calculated an AUC with 1000 bootstrap replicates. Also, to assess the performance with high sensitivity and high specificity, we tested the partial AUC (pAUC) with 80 and 100% sensitivity and specificity [50]. On the other hand, a Kappa coefficient of less than 0.4, of 0.4–0.75, or greater than 0.75 indicates poor, moderate, or good reliability, respectively [33].

$$Accuracy=\frac{Number\;of\;true\;positives+true\;negatives}{Number\;of\;true\;positives+true\;negatives+false\;positives+false\;negatives}$$

$$Sensitivity=\frac{Number\;of\;true\;positives}{Number\;of\;true\;positives+false\;negatives}$$

$$Specificity=\frac{Number\;of\;true\;negatives}{Number\;of\;true\;negatives+false\;positives}$$

$$Positive\;predictive\;value=\frac{Number\;of\;true\;positives}{Number\;of\;true\;positives+false\;positives}$$

$$Negative\;predictive\;value=\frac{Number\;of\;true\;negatives}{Number\;of\;true\;negatives+false\;negatives}$$

$$Kappa\;coefficient=\frac{\left(Percent\;agreement\;observed\right)-(percent\;agreement\;expected\;by\;chance\;alone)}{100\%-(percent\;agreement\;expected\;by\;chance\;alone)}$$

To further evaluate the effect of different cancer stages and other external factors on the diagnostic accuracy, we conducted subgroup analysis and sensitivity analysis with the same statistical method to calculate the indicators and estimated each ellipse of the ROC curves to compare the effect. In subgroup analysis, we used combined subgroups of controls with cases from stage 0 and 1, stage 2 and 3, and stage 4, respectively. In the sensitivity analysis, we excluded subjects with a specific medical history and health behaviors.

### 2.7. Sample Size Estimation

We used the formula for standard error to calculate the sample size required for this study [45]:

$$Standard\;error=\sqrt{\frac{C(100-C)}{N}}$$

where C was the percentage of correct classifications and N was the estimated sample size. With the standard error limited to no more than 5% and aiming for an accuracy rate exceeding 90%, we calculated that the training set requires at least 36 samples.

## 3. Results

**Figure 4:**
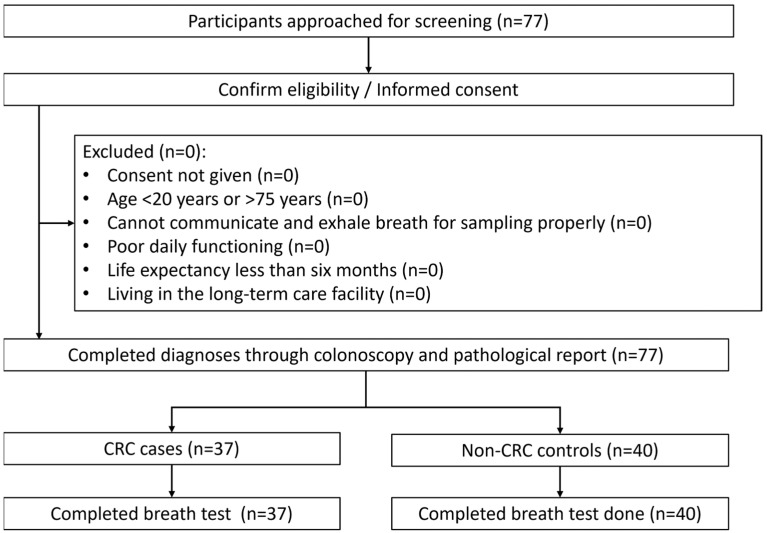
Flow of participants. CRC: colorectal cancer.

A total of 77 subjects were enrolled in this study, with 37 CRC cases and 40 non-CRC controls included. The process of excluding study subjects is provided in Figure 4. The distribution of males was nearly identical in both groups. The cases were older compared to the controls at a statistically significant level. In the cases, subjects were distributed across all cancer stages, with the highest proportion at an early stage (stage 0 and 1). In the controls, most subjects had hemorrhoids while a smaller proportion had benign polyps. There was a slightly higher prevalence of diabetes in the cases, but other medical histories of conditions like kidney disease and COPD were rare in both groups. There was a higher proportion of smokers and drinkers in the cases (Table 1). The time interval between the date of the reference test and breath testing ranged from 5 to 49 days, and the mean time was 20.1 days, with no intervention performed during this time and no adverse events occurring after either test. The sampling bag filled with the exhaled breath was kept for 9 days on average before being sent to the College of Public Health of National Taiwan University for analyzing.

### 3.1. Performance of Predictive Models

In the internal validation, the LOOCV accuracy and Kappa coefficient of the predictive model built using the SVM algorithm with a linear kernel were the highest, so we used this model in the subsequent analyses. We then classified the subjects according to whether they had CRC or not and estimated the overall performance (Table 2). The accuracy was 0.83 (95% CI 0.73–0.91), the sensitivity was 0.81, the specificity was 0.85, the positive predictive value (PPV) was 0.83, and the negative predictive value (NPV) was 0.82.

**Figure 5:**
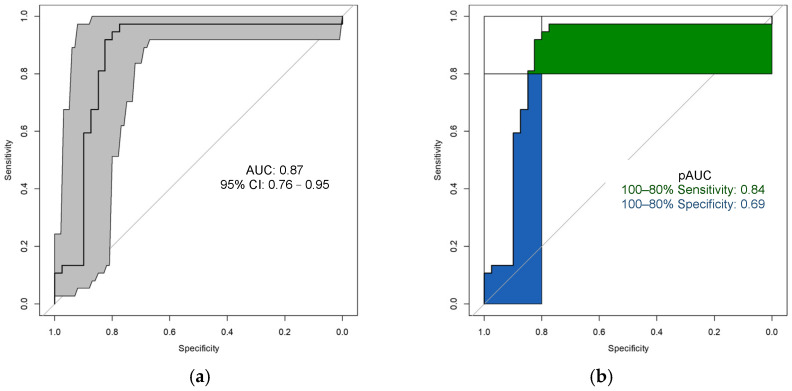
(**a**) The ROC curve and AUC with 95% confidence interval after 1000 bootstrapping replicates of the predictive model classifying the subjects as having CRC or not. The black line is the ROC curve, which illustrates the trade-off between the sensitivity and false positive rate across different thresholds. The gray region shows the 95% confidence interval around the mean curve. (**b**) The pAUC of the predictive model between 80 and 100% for sensitivity and specificity, which corresponds to the green region and blue region, respectively. AUC: area under the ROC curve, CI: confidence interval, pAUC: partial AUC.

The ROC curve, which is shown in Figure 5, with 1000 bootstrapping replicates showed that the 95% confidence interval and the overall AUC to assess the diagnostic accuracy was 0.87 (95% CI 0.77–0.95). The pAUC between 80 and 100% for the sensitivity was 0.84 and the pAUC for the specificity was 0.69. As for assessing the reliability, the Kappa coefficient was 0.66 (95% CI 0.49–0.83). This result indicated that the predictive model had a very good diagnostic accuracy and a moderate reliability.

### 3.2. Subgroup Analysis

**Figure 6:**
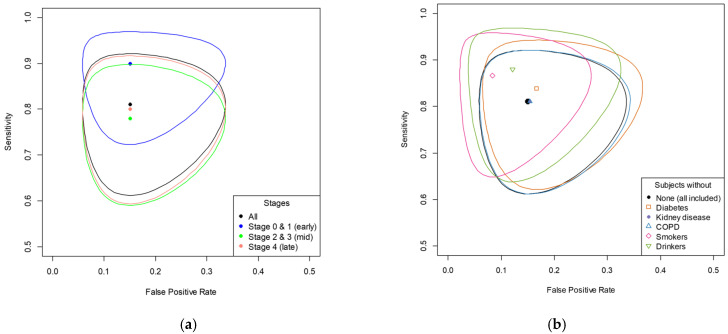
The ROC curve from the subgroup analysis (evaluating diagnostic performance across different stages of disease progression) and sensitivity analysis (assessing the impact of external factors on diagnostic performance). (**a**) Comparison of subgroup analysis of cases in early, mid, and late stage. The color of the points corresponds to a specific subgroup population divided by cancer stages. (**b**) Comparison of sensitivity analysis of exclusion of subjects with potential confounding factors including medical history and health behaviors. The color of the points corresponds to a population that was without specific external factors. Data points closer to the upper left corner of the ROC curve indicate higher diagnostic accuracy [51]. Each ellipse showed the 95% confidence interval, indicating the variability in the sensitivity and false positive rate. COPD: chronic obstructive pulmonary disease.

To assess the diagnostic performance at different stages of disease progression, we compared the ROC curve and its ellipse while classifying the subjects as in the early, mid, or late stage. The diagnostic accuracy of the electronic nose for CRC is better in the early stages. As shown in Figure 6, the ROC curve from the subgroup analysis indicates that the blue point and ellipse, representing the early stages (0 and 1), demonstrate the highest accuracy compared to the green point and ellipse for the middle stages and the red point and ellipse for the late stages. The accuracy ranged from 0.82 to 0.88 and the Kappa coefficient ranged from 0.63 to 0.75, which are both provided in Table S2 along with the sensitivity and specificity for each subgroup.

### 3.3. Sensitivity Analysis

To assess the effect of external factors on the diagnostic performance, we compared the ROC curve and its ellipse while classifying the subjects, excluding those with diabetes (n = 10), kidney disease (n = 1), or COPD (n = 1); those who smoked cigarettes (n = 11); and those who consumed alcohol (n = 19). The results showed that the exclusion of the study subjects with kidney disease and COPD had no significant effect. The exclusion of the subjects with diabetes had a moderate effect. The exclusion of the subjects that smoked cigarettes and consumed alcohol had a significant effect, with the highest sensitivity and specificity derived with excluding the subjects who smoked. The accuracy ranged from 0.83 to 0.90 and the Kappa coefficient ranged from 0.66 to 0.79, and both are provided in Table S3 along with the sensitivity and specificity.

## 4. Discussion

### 4.1. Main Contribution of the Study

This study provides stage-specific sensitivity data for the electronic nose in CRC detection. Our findings indicate that the electronic nose achieves a high accuracy in detecting early-stage CRC, addressing a critical limitation of FIT, which has reduced sensitivity at early stages. By demonstrating that integrating electronic nose breath analysis into existing CRC screening frameworks can significantly enhance early-stage detection, this study highlights its potential to improve the overall effectiveness of screening programs.

### 4.2. Strengths of the Study

We followed the Standards for Reporting of Diagnostic Accuracy (STARD) guidelines to enhance the quality of this study. The detailed requirements of the guidelines are shown in Table S2. This promoted the transparency and comprehensiveness of the details included in our study design for evaluating the electronic nose as an auxiliary screening tool [52]. We used pathological reports with colonoscopies as the reference standard for determining cases in this study, ensuring that each diagnosis of CRC was clearly and accurately determined, which minimized the risk of misclassification bias. Hence, the breath samples from the two groups were validated for comparing breath testing, developing predictive models, and assessing the diagnostic performance.

The sampling and analyzing methods were standardized before the analysis in order to ensure the reproducibility of the results. To capture endogenous VOCs with physiological significance, we ensured that the sampled breath originated from the alveoli [16,17], with the device controlling conditional airflow. To avoid potential variability such as changes in environmental conditions and background odors [45], we established a standardized research process from sampling to analyzing, conducting the entire process in the same room with the same device. Ensuring the reproducibility of the breath testing results remains an essential goal that must be achieved before the electronic nose can be applied in clinical practice [28]. The measurements of the electronic nose used in this study were confirmed with high consistency. We assessed the intraclass correlation coefficient (ICC) to determine the consistency of the measurements 10 times [53,54]. For each subject, the 32 sensors had an ICC of 1, and for each sensor, the 10 randomly sampled subjects had an ICC of 0.99, both indicating that the sensor array in the electronic nose was highly stable and reliable across the individuals and repeated measurements.

### 4.3. Applicability of Findings

The sensitivity and specificity of the predictive model in this study were 0.81 and 0.85, which aligned with the results reported in previous studies. Scheepers et al. reported a sensitivity of 0.93 and the specificity of 0.89 for various cancers [22], and Wang et al. showed a sensitivity of 0.87 and specificity of 0.78 [25], respectively, in a meta-analysis. We additionally reported a pAUC between 80% and 100% for the sensitivity in order to highlight the potential of testing methods applied in populations with a high prevalence of specific disease, such as in clinical settings. The pAUC provides a good indication of the corrected AUC to assess the generalizability of the result, which demonstrates the diagnostic performance without bias [32,45]. Its clinical application was also shown in other pilot study, assisting in the monitoring of CRC recurrence or metastasis [24].

Breath testing using the electronic nose had a better overall performance to detect potential cases through machine learning algorithms, which aligned with the results reported in previous studies [55]. In this study, we additionally provided the diagnostic performance in early-stage CRCs, highlighting the importance of early detection in improving the current status of CRC management [4], emphasizing its value of a powerful method in screening CRC. In the study by Tyagi, H. et al., octanol, nonanal, decanal, and other compounds in urinary headspace VOCs showed statistically significant differences between subjects in the early and late stages of disease [56], supporting this finding. The higher sensitivity observed in the subgroup analysis of this study might be attributed to the relatively less complex pathological changes, which made it easier for the electronic nose to identify VOC compositions associated with CRC progression. While their study reported higher AUCs when distinguishing late-stage CRCs from non-CRCs compared to early-stage CRCs [56], we found a higher AUC when distinguishing early-stage CRCs from non-CRCs. This indicated the potential of early detection through breath testing.

The subjects at an undetermined stage of CRC in this study appeared to have no effect on the results. As shown in Figure S4, the confidence interval largely overlapped with that of other groups, indicating that the inclusion of subjects at an undetermined stage did not introduce bias or variability into the analyses.

Health behaviors and comorbid conditions can affect the performance of the predictive model. Endogenous VOCs were interfered with by individual differences [22,25,57,58]. By excluding subjects that smoked cigarettes and consumed alcohol [42,43] and subjects with diabetes, we observed an improvement in the accuracy and reliability. We believe that the main confounding factor was smoking [42], as the effect of alcohol on metabolic changes seems to occur for a short period of time [43]. In future applications of breath testing, external factors such as smoking should be noted to ensure robustness.

Exploring the comparison of breath testing using an electronic nose and FIT, we found that the specificity was similar, but the sensitivity for early-stage disease was higher than the results provided in a previous meta-analysis [59]. The gradient sensitivity observed in FIT is believed to result from more advanced disease stages, which typically lead to stronger intestinal bleeding. This also indicated the potential of early detection through breath testing compared to FIT. In the study by Imperiale et al., a low sensitivity of FIT in advanced adenomas was reported [60], while in the study by van Keulen et al., the sensitivity of breath testing in advanced adenomas was higher [21], suggesting that methods of discerning the VOC composition may have advantages in identifying metabolic changes during the early stages of CRC progression over undergoing a single round of FIT.

In addition to its potential to improve current screening programs for CRC through reducing false positive results and enabling early-stage detection, breath testing provides the benefit of non-invasive multi-validation. The follow-up rate after positive FIT was suboptimal in Asian programs [2], which was likely due to its invasiveness. Moreover, non-adherence to referral colonoscopies may increase the risk of mortality [61,62]. By integrating breath testing into the two-step approach of FIT and colonoscopy, it could provide complementary information to help individuals be more confident in the decision to proceed with follow-up treatments, enhancing adherence to referral colonoscopies. The overall effectiveness of CRC screening programs could be improved, ensuring the accurate identification of high-risk individuals and making full use of medical resources.

### 4.4. Limitation

Due to the limited sample size, some of the observed differences in the analyses were not statistically significant. The diagnostic accuracy and reliability reported in this study might be decreased. In addition, we did not plan on dividing our dataset into a testing set for external validation. Although the internal validation conducted with LOOCV and with all data showing good results, this decision might present limitations in terms of the generalizability. Before integration into actual clinical practice, there is still a need for validation through larger sample sizes and external data from different populations [32]. While the electronic nose could analyze the VOC composition in exhaled breath, it could not identify specific VOC molecules. This limitation brings about a challenge as medical treatments usually require the quantitative measurement of biomarkers.

### 4.5. Future Directions

To address this study’s limitations, future research should focus on larger and more diverse sample sizes to improve the statistical power and the reliability of the diagnostic accuracy estimates. External validation using independent datasets from different populations is critical for enhancing the generalizability of the findings. Multicenter prospective studies are needed to reduce the selection bias and validate the results in real-world clinical settings. Longitudinal studies are also recommended to assess the diagnostic accuracy of the electronic nose over time, particularly for early-stage colorectal cancer detection. Investigating the underlying physiological mechanisms of VOC production, alongside advancements in sensor technology, will strengthen clinicians’ confidence in sensor-generated results. This approach enables clinicians to integrate VOC data with patients’ pathology, facilitating more precise and informed clinical decisions.

## 5. Conclusions

This study highlights the promising potential of breath testing using an electronic nose for enabling early detection and reducing unnecessary treatments, demonstrating that integrating such a non-invasive method could complement current screening programs, by addressing key limitations such as low sensitivity in detecting early-stage CRCs, false positive results, and non-adherence to follow-up treatments. However, the predictive model requires more independent data for external validation to ensure applicability and generalizability before it can be implemented in clinical practice.

## Figures and Tables

**Table 1:** Characteristics of the study subjects.

Characteristics	CRC Cases (n = 37)	Non-CRC Controls (n = 40)	*p*-Value
Sex (male) (%)	21 (56.76)	23 (57.50)	0.95
Age (mean) (SD)	63.08 (8.09)	52.28 (12.21)	<0.05
Cancer stage (n = 37)			
0 (%)	4 (10.81)	-	-
I (%)	10 (27.03)	-	-
II (%)	6 (16.22)	-	-
III (%)	5 (13.51)	-	-
IV (%)	4 (8.10)	-	-
Others (%) †	14 (10.81)	-	-
Pathology state (n = 37)			
8010 (%)	2 (5.41)	-	-
8140 (%)	32 (86.49)	-	-
8211 (%)	1 (2.70)	-	-
8240 (%)	1 (2.70)	-	-
8263 (%)	1 (2.70)	-	-
Non-cancerous status (n = 40)			
Polyp (%)	-	10 (25.00)	-
Hemorrhoid (%)	-	27 (67.50)	-
No abnormalities (%)	-	3 (7.50)	-
Medical history			
Diabetes (%)	6 (16.22)	4 (10.00)	0.51
Kidney disease (%)	0	1 (2.50)	1.00
COPD (%)	0	1 (2.50)	1.00
Health behavior			
Smokers (%)	7 (18.92)	4 (10.00)	0.26
Drinkers (%)	12 (32.43)	7 (18.91)	0.13

COPD: chronic obstructive pulmonary disease. † The cancer stages of the 14 cases were determined at external hospitals.

**Table 2:** Contingency table of prediction from breath testing and actual diagnosis.

Breath Testing	Pathological Report and Colonoscopy		
	Positive	Negative	Total
Positive	30	6	36
Negative	7	34	41
Total	37	40	77

## Data Availability

De-identified volatile data are available upon request due to privacy and ethical restrictions.

## Supplementary Materials

The following supporting information can be downloaded at https://www.mdpi.com/article/10.3390/bios15020082/s1: Table S1: STARD-2015 checklists. Table S2: Model performance in subgroup analysis by cancer stages. Table S3: Model performance in sensitivity analysis excluding confounding factors. Table S4: Comparative table of the electronic nose and other diagnostic techniques for colorectal cancer screening. Figure S1: Comparison of sensitivity analysis of exclusion of different cancer stages.
